# The Benefits and Barriers of Using Point-of-Care Ultrasound in Primary Healthcare in the United States

**DOI:** 10.7759/cureus.28373

**Published:** 2022-08-25

**Authors:** Keila G Carrera, Gashaw Hassen, Genesis P Camacho-Leon, Francis Rossitto, Franklin Martinez, Tadesse K Debele

**Affiliations:** 1 Department of Gastroenterology, Hospital Universitario Manuel Nunez Tovar, Maturin, VEN; 2 Internal Medicine, University of Maryland Capital Region Medical Center, Largo, USA; 3 Medicine, Addis Ababa University, Addis Ababa, ETH; 4 Progressive Care, Mercy Medical Center, Baltimore, USA; 5 Medicine and Surgery, Parma University, Parma, ITA; 6 Division of Research and Academic Affairs, Larkin Community Hospital, South Miami, USA; 7 División de Estudios Para Graduados, Facultad de Medicina, Universidad del Zulia, Maracaibo, VEN; 8 Medicine, Universidad de Carabobo, Maracay, VEN; 9 Family Medicine, Midwestern University Arizona College of Osteopathic Medicine, Kingman, USA

**Keywords:** pocus (point of care ultrasound), clinical radiology, medical billing, acute abdomen, us health system, screening tools, medical education, diagnostic ultrasound, medical technology, primary care medicine

## Abstract

An effective healthcare system should embrace practices that enhance overall quality and productivity. Training primary care physicians in Point-of-Care Ultrasound (POCUS) has become part of the processes that improve the quality of patient care and serve to guide the diagnostic impression quickly and effectively. With the purpose of highlighting the applications and challenges of POCUS use in US primary health care, we conducted a narrative review based on PubMed-indexed and Cochrane Library English text publications searched in May-July 2022 using a combination of key terms including point of care ultrasound, primary care, and US healthcare. Many studies have shown that POCUS has a positive impact on fostering medical attention and reducing morbidity, mortality, and healthcare costs. Besides assisting in procedures, POCUS has a head-to-toe application in evaluating inflammatory and infectious conditions, acute abdomen, cardiopulmonary function, musculoskeletal and vascular pathologies. However, its uniform implementation is limited across the US healthcare system due to multitudes of barriers such as lack of training, resource scarcity, and low reimbursement. Training primary care physicians in general and emergency care providers, in particular, is key to scaleup POCUS use. Large size studies are paramount to further explore the effectiveness of POCUS and identify key challenges to its implementation.

## Introduction and background

Primary care is the cornerstone of US health care, representing the first contact with physicians for patients seeking care [1]. The point-of-care ultrasound (POCUS) gained popularity in medical practice with the rise of technology and speedy access to imaging modalities [2]. It has been used as part of patient care with wider applications from reducing diagnostic variables to guiding invasive procedures [3]. Using POCUS in primary health care settings has reduced costs and improved the quality of care provided by trained physicians who can efficiently implement it as a rapid bedside diagnostic tool [4]. 

The history of ultrasound goes back to the late 1940s after World War II when the potential of ultrasound energy in medical diagnosis was recognized. In 1951, the efforts of Douglas Howry, a radiologist, and his engineers, Bliss and Posakony resulted in the creation of a two-dimensional ultrasound scanner. Since then, ultrasound has evolved over the years to become a very helpful tool in clinical radiology [5]. POCUS is a safe and effective form of imaging that aid diagnosis and guide medical procedures. During the coronavirus disease 2019 (COVID-19) pandemic, POCUS was applied to predict the clinical outcomes and conveniently anticipate future ICU admission or the need for supplemental oxygen through risk stratification [6]. Nowadays, ultrasound has become more compact, higher quality, and more affordable, facilitating the growth of POCUS which can easily be performed and interpreted by the clinician at the patient's bedside [7,8]. POCUS can be embraced as an important tool by a general practitioner in medical practice and helps reduce health care costs. A systematic review of POCUS in general practice by Andersen et al. revealed that no patients found using ultrasonography for abdominal pathology as time-consuming, stressful, uncomfortable, or embarrassing. Rather, the majority of patients were satisfied with the procedure which gave them a sense of security and most believe that POCUS should be performed routinely [9].

According to Moore and Copel, POCUS is defined as ultrasonography transported to the patient and performed by the provider in real-time. The clinician can then use real-time dynamic images which allow findings to be clinically correlated with the patient's presenting signs and symptoms [10]. POCUS is much safer providing a reduction of ionizing radiation risks in addition to enhanced cost-effectiveness, and prompt diagnosis for optimal patient care. It can be used by family doctors or any specialized care as a diagnostic, procedural, and screening tool [11]. The purpose of this article is to provide a narrative review based on existing literature on the different applications of POCUS and its impact on primary care.

## Review

Methods

In order to explore and highlight multi-purpose applications and the perceived barriers of POCUS use in US primary health care, a narrative review was conducted using various combinations of terms such as "point of care ultrasound", "primary care", and "US healthcare" to identify relevant articles that were searched on literature databases such as PubMed and Cochrane library in May-July 2022. The inclusion criteria were English text articles with at least two of the keywords were present in the source and the exclusion criteria non-English articles and data older than 20 years. 

Clinical uses of POCUS in primary care

Olgers et al. suggested core uses of POCUS in primary care ranging from 131 through 601 scans per general practitioner per year and are most frequently utilized for abdominal, obstetric, and heart assessments in primary care [9]. Furthermore, Wang et al. 2022 conducted an observational study on 888 adults with non-traumatic acute flank pain which showed the positive impact of POCUS in shortening the length of stay in the hospital by less than three hours approximately and further alleviating emergency department congestion [12]. POCUS is acknowledged by the American College of Physicians for its key role in medical practice [13]. The spectrum of POCUS application in the management of acute abdomen includes-

Vascular Aneurysm

Patients with Abdominal Aortic Aneurysm (AAA) may present to their primary care physician with abdominal, groin, and back pain. Symptoms are mostly associated with colic renal pain. However, patients with high-risk categories, such as smokers or former smokers with less than 5-10 years, male sex, and age between 65-74, should be evaluated further. Ultrasound has demonstrated a 97.7-100% sensitivity and 94.4-100% specificity in previous studies. The ultrasound findings for aortic rupture are mainly free retroperitoneal liquid that is sometimes hard to visualize because of the transducer, however, observing an interruption of the aorta, malformation, clot in the lumen, and leaking of liquid can be verified by Doppler [14]. In such cases, ultrasound is a life-saving tool that helps to orient a patient for prompt surgery. 

Free Intraperitoneal Fluid 

Cirrhotic patients may consult the primary care office for diffuse abdominal pain and can sometimes present with intraperitoneal infection. POCUS not only identifies free fluid easily but also confirms the diagnostic visualization of small to large volumes of intraperitoneal fluid. POCUS can help the clinician to be more assertive about the diagnosis, and, can detect up to 10 ml of free fluid if used by well-trained hands [15]. 

Inflammation and Infection 

Appendix: The diagnosis of acute appendicitis primarily depends on clinical symptoms and physical examination. Although abdominal computed tomography (CT) is the gold standard image for acute appendicitis, POCUS provides a radiation-free diagnostic aid avoiding unnecessary delays. Sharif et al. in 2018 found that the sensitivity of POCUS in the diagnosis of appendicitis was 69.2%, and the specificity was 90.6%. POCUS maximizes the diagnostic margin further decreasing the morbidity, mortality, and diagnostic delay on the way to the emergency room [16].

Gallbladder: Ultrasound can detect > 3mm of gallstone becoming an excellent tool for the diagnosis of cholelithiasis. Finding pain over the right upper quadrant (RUQ) during POCUS while compressing the abdomen can be taken as a positive Murphy’s sign [17]. Zenobii et al. compared the sensitivity and specificity of ultrasound (82% and 81%) to magnetic resonance (86% and 82%), Cholescintigraphy (94% and 90%), and computed tomography (94% and 94%) for acute cholecystitis respectively [18]. 

Kidney and bladder: POCUS has been used for acute kidney injuries in inpatient and outpatient settings [19]. Performing an ultrasound promptly has shown better outcomes in patient management with acute kidney injuries who visit the primary care office. POCUS can rule out causes of acute kidney injury (AKI) by demonstrating stone, hydronephrosis, abscess, or inflammation (pyelonephritis). POCUS has a sensitivity of 90% and specificity of 100% in the diagnosis of renal tract obstruction [20]. 

Obstetric and gynecologic conditions: POCUS has been used in obstetrics for several years, but its use in the primary care office is limited to the exploration of various causes of acute abdomen in female patients. Finding ovarian cysts in acute & diffuse abdominal pain helps to provide better care for the patient and avoid unnecessary delays in the surgical intervention [17].

POCUS and other clinical applications 

POCUS is not limited to acute abdomen pathologies. It has also been implemented in other parts such as the head, neck, eyes, soft tissues, and the musculoskeletal system. It is even used as an important utility in the primary care practice to assess the lungs, the heart, and the vessels [21,22]. The head and the neck can be evaluated by POCUS searching for sinuses, great vessels, and thyroid pathologies. Besides, ultrasound helping to guide a central venous line insertion decreases the risk of complications [22].

On the other hand, POCUS gives a fast view of the heart and lungs addressing the cause of some conditions to aim for prompt treatment. It is a valuable instrument in the assessment of the normal function of some organs like the heart, exhibiting the range of left ventricular ejection fraction. The common findings in the heart are left ventricular dysfunction, valvular regurgitation, pericardial disease, and chamber enlargement; and for the lungs are pleural effusion, pneumothorax, interstitial and alveolar processes as well as provide aid in some procedures like thoracentesis [22,23].

POCUS can assist in the observation of soft tissue pathologies such as signs of infection like cellulitis and abscess. Further evaluation of the vascular system by searching for occlusions like deep vein thrombosis (DVT) and reducing the risk of complications such as pulmonary embolism (PE) with quick and appropriate intervention are irrefutable advantages. In the nervous system, it can be applied to detect conditions that can affect the optic nerve sheath diameter and the carpal tunnel [22]. POCUS is used to help exclude some differential diagnoses, guide treatment, and track the clinical progress of illnesses in addition to routine imaging in the primary care setting [21]. 

Importance of POCUS training in primary care

In a pilot, randomized research with 60 patients by Ben-Baruch Golan et al. found that POCUS use resulted in a 5-hour reduction in treatment time compared to a 24-hour control group for patients admitted to internal wards with chest discomfort or dyspnea [24]. Numerous studies have emphasized the value of POCUS; for instance, a study performed on 58 internal medicine residents found that 84 % of them were able to obtain independently high-quality images from the abdominal aorta and kidney and 98% reported an increase in confidence with ultrasound use [25].

The American Academy of Family Physicians (AAFP) has suggested guidelines for family medicine residency programs to implement a curriculum in POCUS [17]. Also, the American College of Physicians recognized the importance of POCUS in internal medicine practice proposing future guidelines on generalists and subspecialties [13]. The importance of integrating POCUS into internal medicine and family medicine curriculum is expected to increase shortly. 

Challenges of using POCUS in primary care 

POCUS in primary care has been controversial due to its common use in other specialties such as emergency, and surgery, where a prompt diagnosis is vital to decide quick management. Although, the implementation of POCUS in primary care has brought some benefits like increasing precision of diagnosis during the visit and therefore, appropriate treatment options. POCUS can facilitate the recognition of some illnesses at a lower cost and also prevent unnecessary further studies and management [26]. Even though applying POCUS in primary care can be very helpful in narrowing down the differential diagnoses related to the specific signs and symptoms presented by the patient during a primary care consult, there are some pitfalls that should be considered.

The primary care physician should know how to use and interpret POCUS in order to avoid equivocal and uncertain findings. Not all residents are trained to read POCUS during an internal medicine or family medicine residency as it is not a requirement from the Accreditation Council for Graduate Medical Education (ACGME). In addition, the sensitivity and specificity of POCUS sometimes rely on the severity of the disease. Performing POCUS in every consult might require extra time to spend and further adjustment of the daily schedule which might reduce the number of patients seen daily in the primary care office [27].

A survey conducted by the University of Vermont demonstrated that only 5% of family medicine providers are using POCUS in their practice but 78% would like to use it, as well as 70% of providers believe that POCUS increases health care delivery [28]. In summary, many challenges are present in the use of POCUS such as a lack of training, time-consuming application, reimbursement issues, and equipment costs.

Billings for ultrasound use 

Clinical ultrasound has become an essential tool that improves diagnosis and patient management. However, one of the barriers to its implementation in the US health care system is attributed to the lower rate of billing (9.3% ) [29]. The ultrasound performed by providers is included within current procedure terminology (CPT) for billing. Sometimes, billing ultrasound procedures becomes complicated as some codes are still not clear for evaluation in some diseases and the same ultrasound can be performed in the same organ for follow-up care [30].

## Conclusions

POCUS is a life-saving bedside technology with a broader application for diagnostic, procedural, and screening purposes. Narrowing down diagnoses and shortening hospital stays during emergency visits are key advantages contributing to reduced morbidity, mortality, and health care costs. Lack of POCUS training, high cost of ultrasound machines, low reimbursement, and the need for dedicated time for imaging and interpretation which may ultimately slow down the patient flow in primary care offices are some of the most common challenges. Formal training of medical students, residents, and practicing physicians helps to maximize the benefits of POCUS use. Integrating POCUS into the primary care setting ensures the quality of care, the safety of patients, and the openness to medical technology. More collaboration with stakeholders and continuously researching evidence-based care are key to implementing POCUS in primary practice. 
